# Thymol protects against 6-hydroxydopamine-induced neurotoxicity in in vivo and in vitro model of Parkinson’s disease via inhibiting oxidative stress

**DOI:** 10.1186/s12906-022-03524-1

**Published:** 2022-02-10

**Authors:** Saeideh Nourmohammadi, Sanaz Yousefi, Mahboubeh Manouchehrabadi, Mona Farhadi, Zahra Azizi, Anahita Torkaman-Boutorabi

**Affiliations:** 1 Department of Microbiology, Karaj Branch, Islamic Azad University, Karaj, Iran; 2 Research Center for Cognitive and Behavioral Sciences, Tehran University of Medical Sciences, Tehran, Iran; 3 Department of Physiology and Pharmacology, Pasteur Institute of Iran, Tehran, Iran; 4 Department of Neuroscience and Addiction Studies, School of Advanced Technologies in Medicine, Tehran University of Medical Sciences, Tehran, Iran

**Keywords:** 6-Hydroxydopamine, Neuroprotection, PD, PC12, Thymol, Rats

## Abstract

**Background:**

Parkinson’s disease (PD) is a multifactorial movement disorder with the progressive degeneration of the nigrostriatal system that impairs patients’ movement ability. Oxidative stress has been found to affect the etiology and pathogenesis of PD. Thymol, a monoterpenic phenol, is one of the most important dietary constituents in thyme species. It has been used in traditional medicine and possesses some properties including antioxidant, free radical scavenging, anti-inflammatory. In this study, in vitro and in vivo experiments were performed with the thymol in order to investigate its potential neuroprotective effects in models of PD.

**Methods:**

The present study aimed to evaluate the therapeutic potential of thymol in 6-hydroxydopamine (6-OHDA)-induced cellular and animal models of PD.

**Results:**

Post-treatment with thymol in vitro was found to protect PC12 cells from toxicity induced by 6-OHDA administration in a dose-dependent manner by (1) increasing cell viability and (2) reduction in intracellular reactive oxygen species, intracellular lipid peroxidation, and annexin-positive cells. In vivo, post-treatment with thymol was protective against neurodegenerative phenotypes associated with systemic administration of 6-OHDA. Results indicated that thymol improved the locomotor activity, catalepsy, akinesia, bradykinesia, and motor coordination and reduced the apomorphine-caused rotation in 6-OHDA-stimulated rats. Increased level of reduced glutathione content and a decreased level of MDA (malondialdehyde) in striatum were observed in the 6-OHDA rats post-treated with thymol.

**Conclusions:**

Collectively, our findings suggest that thymol exerts protective effects, possibly related to an anti-oxidation mechanism, in these in vitro and in vivo models of Parkinson’s disease.

## Background

After Alzheimer's disease, Parkinson's disease (PD) is known as the second most common neurodegenerative disorder, with irreversible loss of dopaminergic (DA) neurons in the substantia nigra pars compacta being its major characteristic [1, 2]. Although recent research has advanced the understanding of how the cascade of degenerative events ends up with cell death, gaps remain in the trigger for PD. Studies identify a common oxidative-damage pathway for the etiology of diseases with presentations similar to PD [3–5], implying that compounds with interference in the generation of reactive oxygen species (ROS) and nitric oxide (NO) or with deterioration in mitochondrial activity are more likely to have a protective role [6]. Furthermore, there are some in vivo and clinical reports supporting the effect of oxygen-free radicals and oxidative stress on the pathogenesis of the disease [7–9]. The presence of 6-hydroxydopamine (6-OHDA), which is a hydroxylated analog of dopamine [10], can cause large oxidative stress and consequently impair DA neurons both in vitro and in vivo [11–13].

Despite the high prevalence of neurodegenerative diseases as well as the large-scale research designed to explore the underlying molecular mechanisms, it is still unclear why none of the currently available medications are effective in slowing or stopping the symptoms of PD [14, 15]. Indeed, the Food and Drug Administration (FDA) approved drugs have been mostly found with limited efficacy in controlling PD symptoms like changes in movement capabilities [16]. To overcome such issues, medicinal plants hold promise for preventing the disease, and current evidence indicates that products of plant origin carry neuroprotective effects [17–19].

**Fig. 1:**
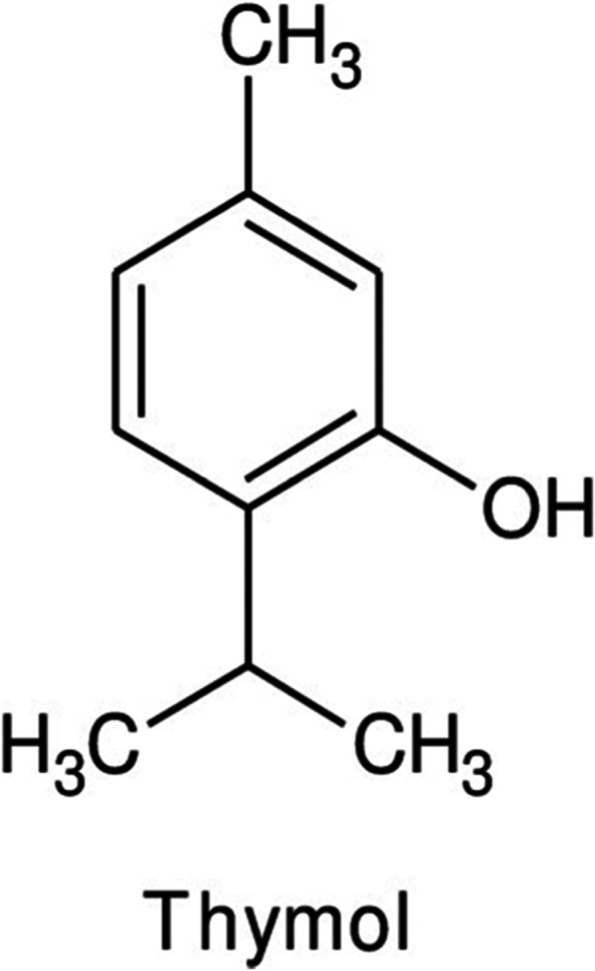
Structure of Thymol [2-isopropyl-5-methylphenol]

Cellular homeostasis necessitates a balance between pro-oxidants and antioxidants. Therefore, attempts to restore the cellular antioxidant system are potential therapeutic approaches whereby vulnerable dopamine (DA) neurons are protected from oxidative stress and further inflammation. But, concern has been raised about the adverse effects of anti-inflammatory agents and the pro-oxidant actions of synthetic antioxidants. In this way, plant extracts and phytochemicals provide a better basis for therapeutic and preventive purposes in PD while having antioxidant and anti-inflammatory activities [20, 21]. Accordingly, phytochemicals with great antioxidant and anti-inflammatory effects and lesser cytotoxicity are becoming the main focus of pharmacological therapy [21]. Thymol, a well-known dietary phytochemical, has been popular because of its desired physicochemical and pharmacokinetic characteristics [22]. It is a monoterpene (2-isopropyl-5-methylphenol) (Fig. 1) mainly present in a large number of edible or culinary plants and plays active roles as an antioxidant, anti-inflammatory, antimutagenic, analgesic, and anti-microbial agent. This compound has also received approval for culinary, cosmetic, and food industries. Given its dietary use for a long period, favorable safety, and low toxicity, it is of interest in neurodegenerative diseases to investigate its possible contribution. Hence, the present study was intended to examine the neuroprotective efficacy of thymol and underlying mechanism in 6-OHDA-induced cellular and rat models of neurodegeneration—mimicking PD in humans.

## Material and methods

### Chemicals

Thymol, 6-OHDA, 3-(4,5-dimethylthiazol-2-yl)-2,5-diphenyl-tetrazoliumbromide (MTT), Hoechst 33,258, propidium iodide (PI), and 2′-7′-dichlorofluorescein diacetate (DCF-DA) were purchased from Sigma-Aldrich (USA). RPMI-1640, fetal bovine serum (FBS), phosphate-buffered saline (PBS), and penicillin/streptomycin were purchased from Gibco (USA). DMSO from Takara (Japan) and thiobarbituric acid (TBARS) from Merck (USA) were used in this study.

### Cell culture and treatment

**Fig. 2:**
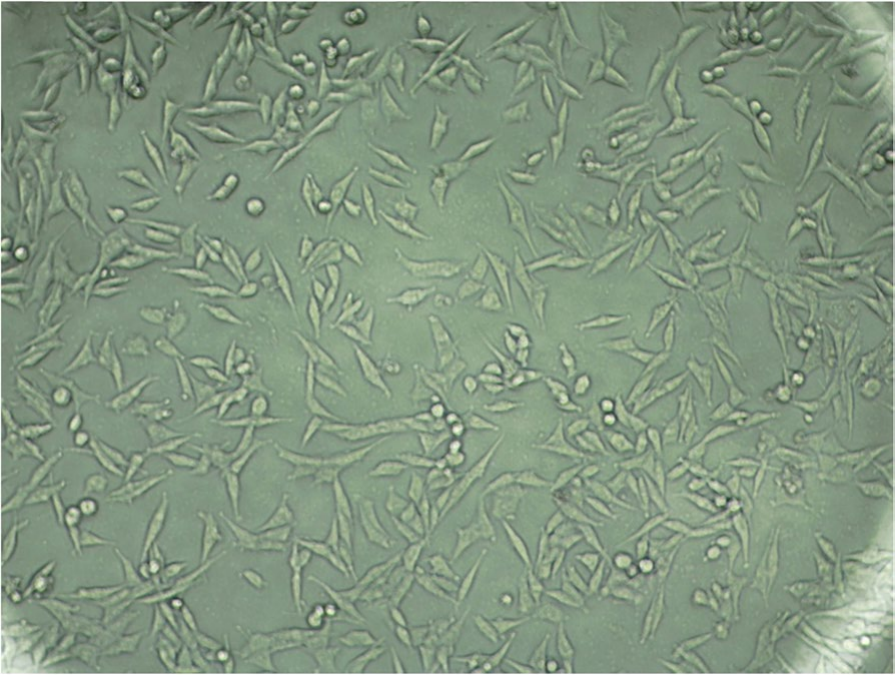
PC12 cell morphology

One cell line widely used in the experimental setting is PC12 cells, a cloned line of pheochromocytoma cells derived from the rat adrenal medulla, which have substrates for the intracellular synthesis, metabolism, and transport of dopamine [11]. Accordingly, this cell line allows for the investigation of PD via the induction of apoptosis by 6-OHDA [23, 24]. PC12 cells were obtained from Pasteur Institute of Iran (Tehran, Iran). The cells were grown in RPMI-1640 supplemented with 10% FBS and 1% penicillin/streptomycin at a humidified atmosphere of 95% air and 5% CO_2_ at 37 °C (Fig. 2). The culture medium was changed every 2–3 days. After the cells reached 70% confluency, they were passaged at a split ratio of 1:3. Then, a density of 5 × 10^3^ cells per well was placed in 96-well plates and exposed to 75 μM of 6-OHDA or thymol (10, 50, 100, 150, and 200 μM) for 24. To evaluate the neuroprotective influence of thymol, the 6-OHDA (75 μM)-treated cells subsequently received thymol at 10, 50, 100, 150, and 200 μM for 24 h [25].

### Determination of cell viability

The MTT assay was conducted to measure the effect of thymol on the viability of the PC12 cells treated with 6-OHDA. The MTT reagent is a yellow tetrazole and undergoes a reduction in the mitochondria of living cells to purple formazan. This water-insoluble crystal was dissolved in DMSO, and absorbance was measured at 570 nm using a microplate reader (BioTek, ELx800, USA) with background correction performed at 690 nm. The cell viability was described as the percentage of the surviving control cells [26].

### Measurement of ROS generation

The quantification of intracellular ROS levels was determined by applying the H_2_DCF-DA probe (Sigma, USA). In brief, 10 μM H_2_DCF-DA was added to the PC12 cells pretreated with 6-OHDA or its combination with thymol under incubation for 1 h at 37 °C in the dark. After resuspending in PBS, the intracellular production of ROS was measured via monitoring the fluorescence intensity of H_2_DCF-DA. The fluorescence reads were taken at an excitation wavelength of 480 nm and an emission wavelength of 530 nm [27].

### Lipid peroxidation assay

24 h after the drug treatment, the cell lysis was performed by scraping in 2.5% (w/v) trichloroacetic acid (TCA). Centrifugation at 10,286 rpm was applied for 2 min at 4° C, and the supernatant (0.5 mL) was collected to be mixed with 0.8 mL of thiobarbituric acid (0.67% (w/v)) and 0.4 mL of TCA (15% (w/v)). The second centrifugation at 2500 rpm was carried out for 10 min at 4 °C. The intensity of fluorescence was detected using a multiwell plate reader. The excitation and emission wavelengths were 530 and 550 nm, respectively [26].

### Hoechst staining of nuclear DNA

The detection of apoptotic nuclei was carried by Hoechst 33,258 staining visualized by fluorescence microscopy. After 24-h treatment with 6-OHDA alone or in combination with thymol, the cells were fixed in 4% paraformaldehyde for half an hour, washed thoroughly three times with PBS, and stained with 3 mg/mL Hoechst 33,258 during 30 min of incubation in the dark at room temperature. The fluorescence microscope allowed the observation of the living cells washed twice with PBS at × 40 magnification (inverted florescence microscope model: IM-3FL4) at 357-nm excitation and 447-nm emission wavelength, respectively. Apoptosis occurred in the cells characterized by decreased nuclear size, chromatin condensation, intense fluorescence, and nuclear fragmentation [26].

### Flow cytometric detection of apoptosis

The further distinction of 6-OHDA-induced cell death modalities, including early apoptosis and late apoptosis/necrosis, was obtained by annexin V/PI staining protocol. Briefly, the pretreated PC12 cells were collected and then resuspended in 100 mL binding buffer. Annexin V (5 mL) and PI (5 mL) solutions were added in the dark at room temperature. After 15 min, the cells were analyzed in a FACS can flow cytometer (Coulter EPICS-XL, Coulter, Miami, FL, USA) applying the Cell Quest software. The FL1 and FL2 channels were used for annexin V and PI, respectively [28].

### Animals

In vivo studies were conducted on 48 male Wistar rats (200 – 250 g) obtained from Pasteur Institute of Iran (Tehran, Iran). The animals were group-housed four per cage in an animal room maintained at 24 ± 1 °C under the normal condition of a 12-h light–dark cycle. All rats were given unrestricted access to food and water. To avoid the potential effect of hormonal variations, we did not use female animals. The animal research and care procedures followed International Guideline for the Care and Use of Laboratory Animals. The study is reported in accordance with ARRIVE guidelines. The Research and Ethics Committee of Tehran University of Medical Sciences (TUMS) approved the experiments (Date 2019/10/21, IR.TUMS.VCR.REC.1398.641).

Animals’ groups:

- Group 1: Control: surgery without any injection
- Group 2: 6-OHDA: 6-OHDA injection after surgery
- Group 3: tween80: 6-OHDA injection after surgery + tween 80 injection for 14 days
- Group 4: Thymol 20: 6-OHDA injection after surgery + thymol 20 injection for 14 days
- Group 5: Thymol 30: 6-OHDA injection after surgery + thymol 30 injection for 14 days
- Group 6: Thymol 40: 6-OHDA injection after surgery + thymol 40 injection for 14 days

### Surgical procedure and drug treatment

**Fig. 3:**
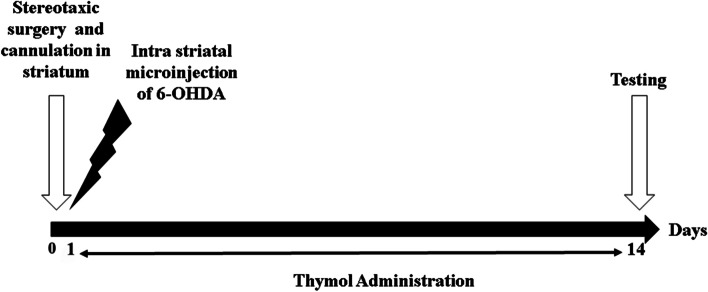
Diagram indicating the time line of the experimental procedures: surgery and testing in 6 groups of animals

Anesthesia was induced in the rats by applying an intraperitoneal injection of ketamine (80 mg/kg) and xylazine (10 mg/kg). After drilling a hole in the skull, and stereotactic coordinates from the bregma (AP, + 0.5; ML, − 2.5; DV, + 5.0) and (AP, − 0.9; ML, − 3.7; DV, + 6.5) were used for the unilateral injection via a microsyringe (Hamilton Bonaduz AG, Bonaduz, Switzerland) fitted with a 26-gauge needle in the caudate-putamen [29]. The rats (*n* = 6) received a 2.5-μL solution containing 6-OHDA (6 μg/μL, Sigma-Aldrich) into each point over 5 min to assure the solution diffusion. 24 h after 6-OHDA microinjection, different doses of thymol (10, 50, 100, 150 and 200 mg/kg) dissolved in saline and tween 80 (10%) were administered intrapertoneally to animals once daily for 14 consecutive days (Fig. 3). On day 15, the following tests were performed, respectively: bar test, pole test, beam walking, rotarod test, open field and apomorphine induced rotation test. The behavioral tests were not video recorded.

### Pole test

Motor coordination was examined using pole test described by Hue et al. with a slight modification [30]. The test included a pole with a height of 50 cm and a diameter of 1 cm, wrapped in gauze to prevent the rats from slipping. The rats were placed on the pole facing their heads upward. The total time taken to reach the floor and the time at which the rat turned head downward were documented. The test was replicated four times for each rat, and the average time for three tests was used for further statistical analyses [31].

### Catalepsy test

The cataleptic state is characterized by fixity of posture as well as difficulty in the initiation of movement. In our study, the catalepsy-like immobility was assessed by placing the rat’s forepaws gently on a bar (0.9-cm diameter and 10 cm above the platform). The test was carried out four times for each rat, and the average time for three tests was used for further statistical analyses [31].

### Beam walking test

A 105-cm length beam apparatus with a flat surface of 4 cm width was employed to examine both symptoms of akinesia and bradykinesia in the rats. The beam was suspended 80 cm above the ground by two supports at both ends. A start line was drawn 20 cm from one end of the beam, while a platform, containing the home cage bedding material of the rat as motivation, was placed at the opposite end of the beam. To determine akinesia time, each rat was positioned completely behind the start line and faced its home cage. The latency time was recorded to step over the start line with all four feet within 1 min. Furthermore, the total time taken to traverse the beam and all four feet entirely upon the finishing platform was considered as bradykinesia time. When a rat failed to reach the home cage within 2 min or fell down, the test was cancelled, and the maximum time was recorded for that trial [31].

### Accelerated rotarod test

The rotarod test was conducted to examine motor coordination and learning ability and balance maintenance. The diameter of rotarod bar was 80 mm. To this aim, 5-min trials were considered to train the rats on the rotarod twice a day for 3 consecutive days. The speed gradually increased from 5 to 15 rpm. On day 15, the rats underwent the first test session with an accelerating speed of 5 – 20 rpm over 20 s and were subsequently tested within the range of 5 – 45 rpm over 200 s. Average latency to fall from the rod was reported as the time each rat could hold itself on the rotating rod. The travel distance was recorded, as well [32].

### Open-field test

Locomotor activity was evaluated using the open-field apparatus. After adaptation, the rats were placed in the center of glass, where they could freely explore the glass for 15 min. The number of crossed squares was reported as a locomotor activity [33].

### Apomorphine-induced rotation

Apomorphine (1 mg/kg in 0.5% ascorbic acid-saline) was injected into the rats intraperitoneally on day 15. The apomorphine-induced rotational test was performed to measure motor asymmetry 5 min after injection. The results are reported as rotation per 30 min.

### Tissue preparation for TBARS and GSH assays

After behavioral testing, the animals were sacrificed with CO2 inhalation and then their brains were immediately removed for collecting striatum. For the assessment of TBARS (Thiobarbituric acid reactive substances) and GSH, the homogenized collected striata were frozen at − 80 °C [32].

### Reduced glutathione

The level of GSH was determined by precipitating of 0.2 mL homogenate with 0.2 mL of sulfosalicylic acid (4%). The mixture was kept for 1 h at 4 °C and then was centrifuged at 1200 × g for 15 min at 4 °C. The assay mixture was filtered and aliquoted into 0.1-mL portions. Then, 1.7 mL phosphate buffer (0.1 M, pH 7.4) and 0.2 mL DTNB (4 mg/1 mL of phosphate buffer, 0.1 M, pH 7.4) were added to filtered aliquot to a total volume of 2 mL. The developed yellow color was read immediately at 412 nm. The results are indicated as nmol GSH/g tissue [34].

### Lipid peroxidation

For the assessment of TBARS, 0.5 mL of homogenate was mixed with 1.5 mL of thiobarbituric acid solution containing TBA (1.25 g), trichloroacetic acid (40 g), and concentrated HCl (6.25 mL) dissolved in 250 mL deionized water (TBA) (4%). The total mixture was placed in 100 °C water bath for 30 min. Then, they were transferred to ice bath for 5 min and centrifuged at 3000 × g for 15 min. the absorbance of the supernatant was read through a spectrophotometer three times for each sample at 532 nm. Malondialdehyde (MDA) concentration was measured by absorbance coefficient of MDA-TBA complex (1.56 × 10^5^ M^−1^ cm^−1)^ TBARS concentration is shown as MDA µmol/g of wet tissue [35].

### Verification of cannula placements

**Fig. 4:**
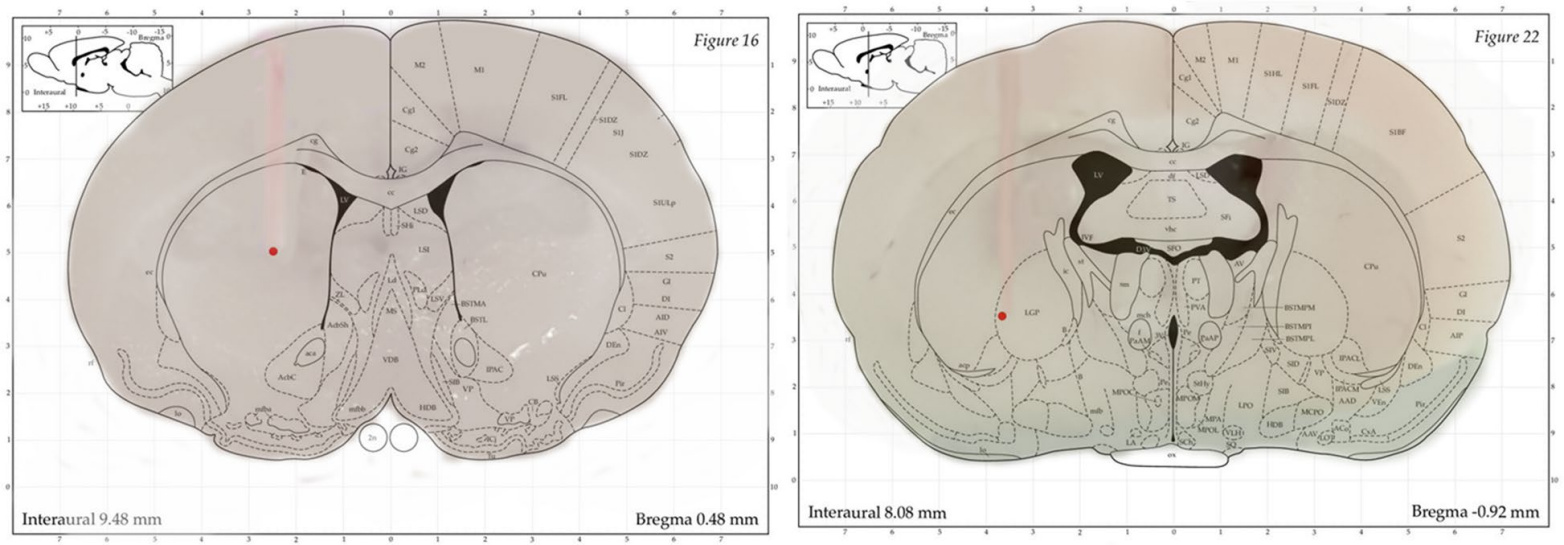
Approximate location of the injection cannula tips in the striatum region

After completing the behavioral studies, the animals were sacrificed with CO2 inhalation. Then, 0.5 μL/side of aqueous methylene blue solution (1%) was injected into the striatum by a 27-gauge injection cannula. Subsequently, the brains were removed and fixed in 10% formalin solution 1 week before sectioning. To assess the location of cannulas pointed to the striatum, sections were evaluated. Histological cannula placement in striatum was verified according to Paxinos and Watson [36] (Fig. 4).

### Statistical analysis

Data are presented as mean ± standard error mean (SEM). The significant difference was determined for multiple groups using one-way analysis of variance (ANOVA) followed by Tukey’s multiple-comparison post-hoc test. In this study, *p* < 0.05 was considered statistically significant.

## Results

### In vitro assay

#### Effect of thymol on cell viability

**Fig. 5:**
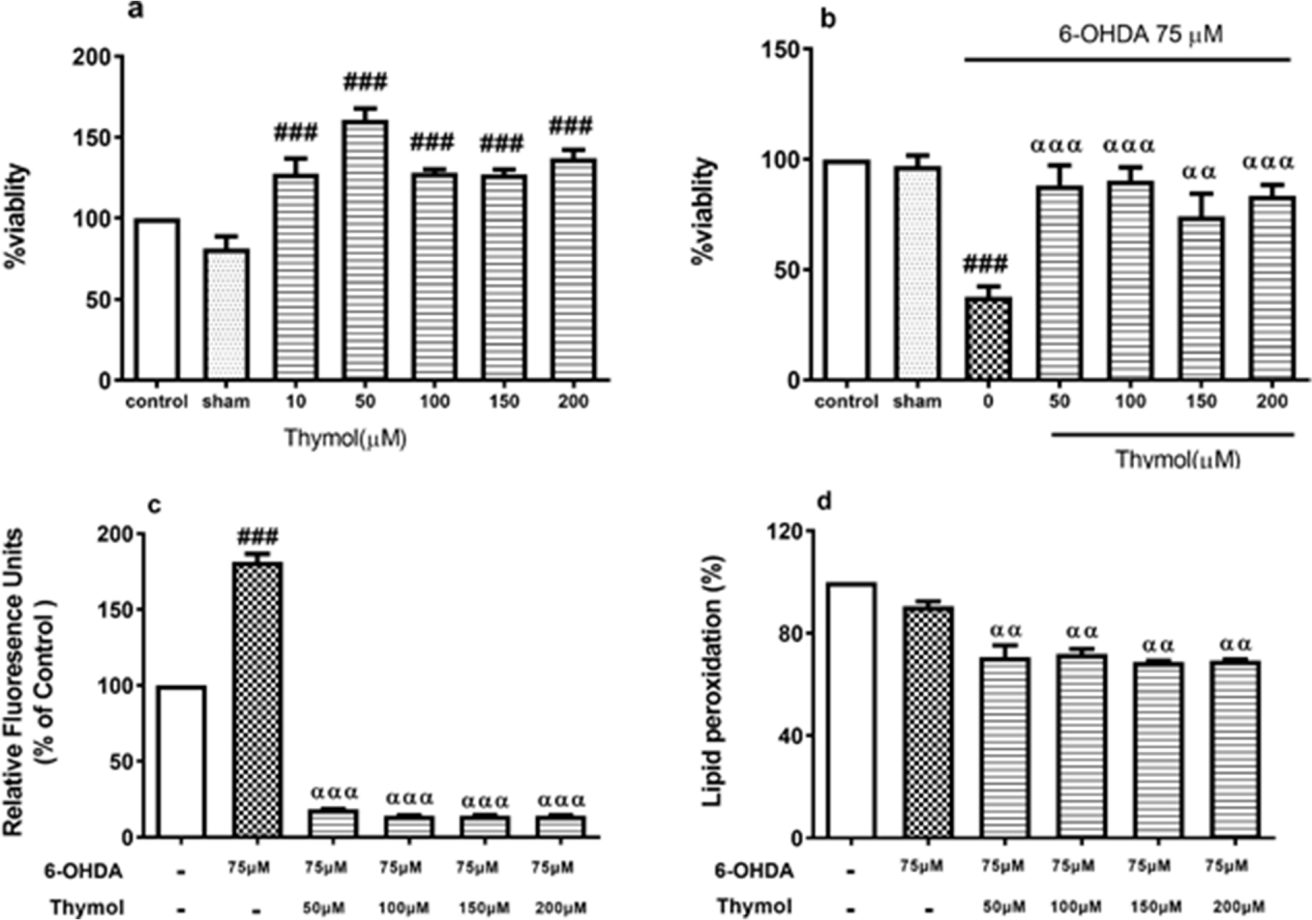
Effects of thymol and 6-OHDA in the presence of thymol on PC12 cells. **a** Effect of different doses of thymol on the viability of PC12 cells for 24 h. **b** Effect of 24-h post-treatment with the different concentrations of thymol on the viability of PC12 cells initially exposed to 6-OHDA for 24 h. **c** Effect of 24-h post-treatment with the different concentrations of thymol on the ROS generation in PC12 cells exposed to 6-OHDA for 24 h. **d** Effect of 24-h post-treatment with the different concentrations of thymol on lipid peroxidation in PC12 cells exposed to 6-OHDA for 24 h. Values shown are means ± SEMs of three independent experiments performed in 4 replicates. ### *p* < 0.001 versus the untreated control cells; αα *p* < 0.01 and ααα *p* < 0.001 versus the 6-OHDA treated cells

As can be seen in Fig. 5a, treatment with different concentrations of thymol significantly increased PC12 cell viability (F(6, 14) = 20.52, *p* < 0.001) in comparison with sham group. The highest growth of PC12 cells was at 50 μM thymol. The possible neuroprotective role of thymol against 6-OHDA was examined by 24-h exposure to 6-OHDA (75 μM) followed by 24-h treatment with thymol (50, 100, 150, and 200 μM). Our previous study showed that the viability reduced to half in the cells treated with 75 μM 6-OHDA, this concentration was used for the present in vitro experiments [37]. Our result revealed that all concentrations of thymol significantly reversed 6-OHDA-induced neurotoxicity (F(6, 35) = 10.70, *p* < 0.001) (Fig. 5b). Thymol at 100 μM mitigated 6-OHDA-induced cell death by more than two folds.

#### Effect of thymol on in vitro 6-OHDA-induced intracellular ROS production

The oxidative conversion of cell-permeable DCF-DA (2’,7’-dichlorofluorescein diacetate) to fluorescent DCF (dichlorodihydro fluorescein) by 6-OHDA (75 μM) was determined as a measurement of ROS generation. In Fig. 5c, it was found that treatment with 6-OHDA led to a significant increase in fluorescence, which was almost 1.5-fold relative to the control group. This effect significantly reduced by thymol at all concentrations. (F(5, 270) = 1041, *p* < 0.001) (Fig. 5c).

#### Effect of thymol on in vitro 6-OHDA-induced lipid peroxidation

Thiobarbituric acid reactive substances (TBARS) assay was carried out to evaluate the anti-lipid peroxidation activity of thymol. Treatment with 6-OHDA had no significant effect on lipid peroxidation in comparison with the control group. However, different doses of thymol could significantly reduce lipid peroxidation (F(5, 6) = 36.12, *p* < 0.001) (Fig. 5d).

#### Effects of thymol on 6-OHDA-induced changes in nuclear morphology

**Fig. 6:**
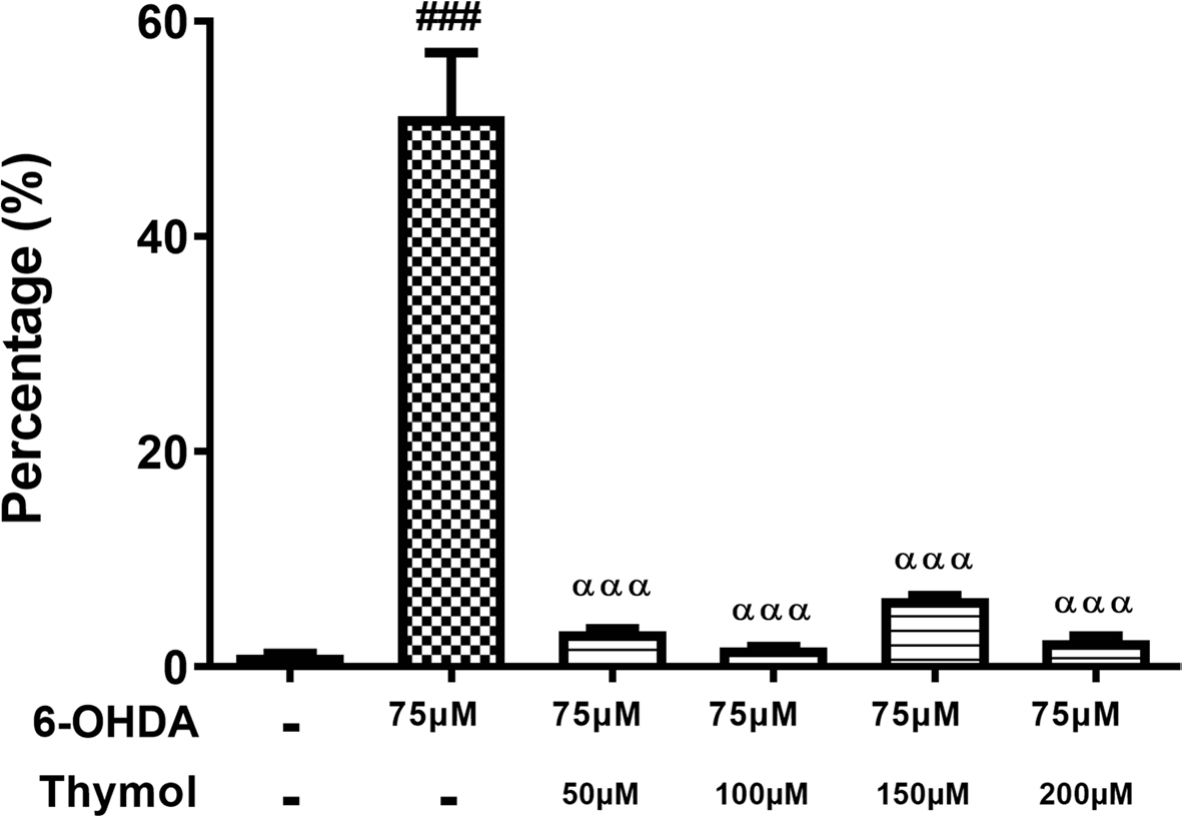
The apoptosis of PC12 cells after treatment with 6-OHDA and 6-OHDA in the presence of different doses of thymol measured by Hoechst 33,258 staining. Each bar represents mean ± SEM; ###*P* < 0.001 versus the control group, ααα *P* < 0.001 versus the 6-OHDA group

Morphological assay of cell death was performed by Hoechst 33,258 staining. In this regard, the normal nucleus was associated with homogeneous staining, presenting regular contours as well as rounded shapes. Following exposure to 6-OHDA (75 μM) for 24 h, most cells produced an asymmetrical, highly bright fluorescence, and the percentage of cells with condensed nuclei significantly increased as compared to the control group (Fig. 6) (F(5, 18) = 66.85, *p* < 0.001). These dramatic changes in the nuclear morphology were prevented significantly by post-treatment of cells with thymol, particularly at 100 μM (Fig. 6).

#### Effects of thymol on percentage of 6-OHDA-induced apoptosis

The Annexin-V^−^ /PI^−^ population consists of normal healthy cells, whereas Annexin-V^+^ /PI^−^ cells appear in the early apoptotic stage, and Annexin-V^+^ /PI^+^ cells are present in the late apoptotic/necrotic stage. Table 1 displays the percentage of apoptosis in PC12 cells. Upon 24 h of incubation with 6-OHDA (75 μM), the percentage of vital cells decreased significantly in comparison to the control group. Thymol at 150 μM showed a positive effect on the percentage of late apoptosis, however, it was not statistically significant.

**Table 1:** Percentage of PC12 cells in each state after treatment with 6-OHDA, 6-OHDA + thymol 100 µM, 6-OHDA + thymol 150 µM. The data presented are mean ± SEM of two independent experiments

**Compound/Concentration**	**Vital cells (%) An-/PI-**	**Early Apoptosis (%) An + /PI-**	**Late apoptosis (%) An + /PI + **	**Necrosis** **(%) An-/PI + **
**Control**	96.05 ± 0.75	0.23 ± 0.05	1.90 ± 0.67	1.64 ± 0.64
**6-OHDA 75 μM**	68.80 ± 3.00^≈^	2.10 ± 0.12	8.4 ± 1.1^∼^	27.80 ± 0.30^≈^
**6-OHDA/Thymol 100 μM**	79.90 ± 1.8	2.31 ± 0.79	5.92 ± 0.88	17.76 ± 2.36
**6-OHDA/Thymol 150 μM**	87.00 ± 2.7^*^	3.01 ± 0.98	6.59 ± 0.61	15.76 ± 2.96^*^

^∼^*p* < 0.05 and ≈ *p* < 0.01 compared with control, *p < 0.05 compared with 6-OHDA

### In vivo assay

#### Effect of thymol on catalepsy test in 6-OHDA-lesioned rats

**Fig. 7:**
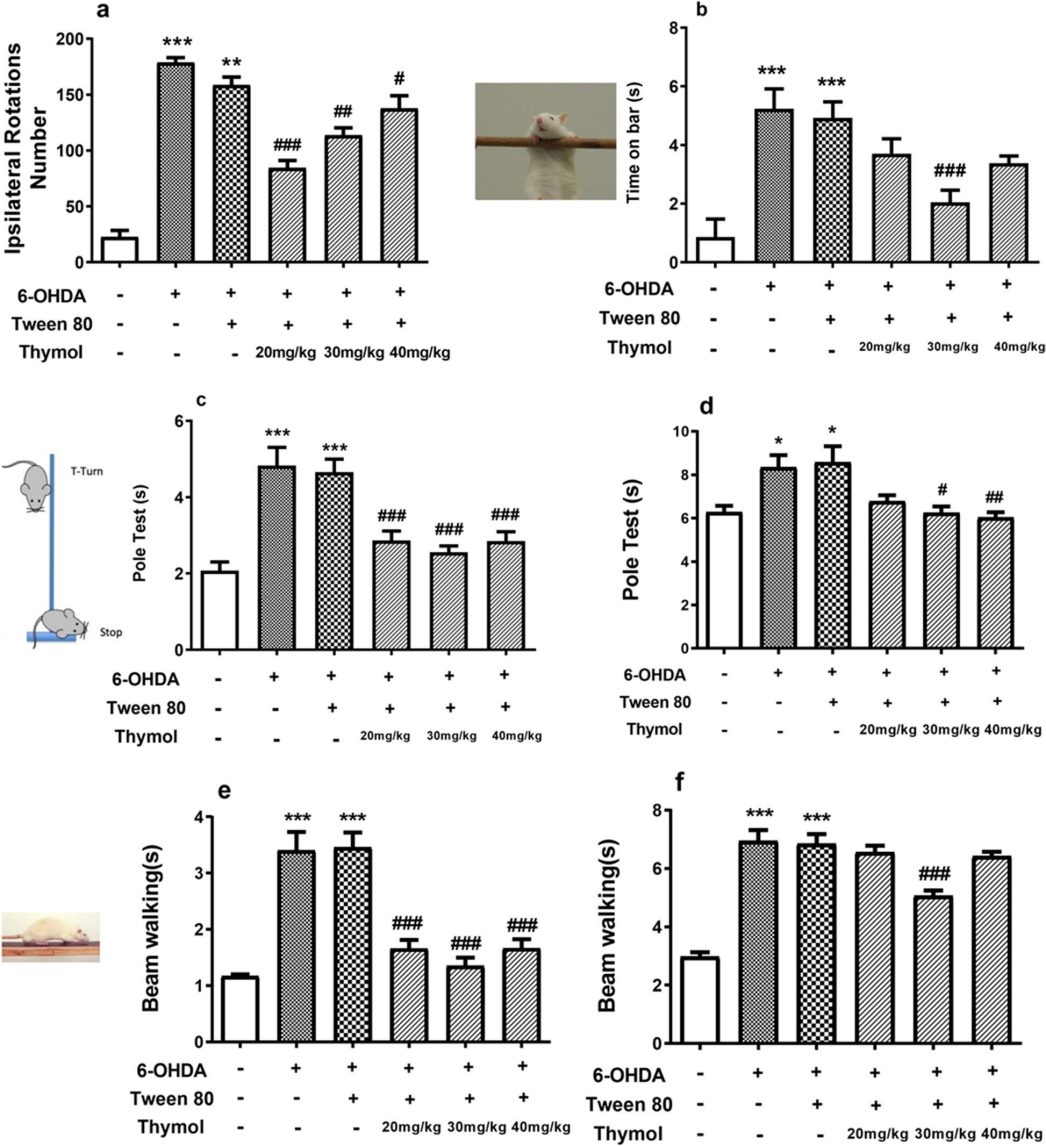
Effects of the intraperitoneal administration of thymol for 15 days on 6- OHDA-induced motor impairment. Apomorphine-induced rotational behavior (**a**), time in the bar test (**b**), motor coordination, and balance in the pole test: both inversion and total time (**c**, **d**) and the beam walking test (**e**, **f**). The values are presented by means ± SEM for six rats in each group. *p < 0.05 and ***p < 0.001 versus the untreated control group; #*p* < 0.05 and ##*p* < 0.01 and ###*p* < 0.001 versus the 6-OHDA-treated groups

The 6-OHDA-treated rats developed muscle rigidity or catalepsy so that the time on bar was significantly increased when compared with that in control group. (*p* < 0.001). Treatment with 30 mg/kg of thymol counteracted such increase. (F(5, 48) = 12.03, *p* < 0.001) and could effectively mitigate 6-OHDA-induced catalepsy (Fig. 7b).

#### Effect of thymol on pole test in 6-OHDA-lesioned rats

The contribution of the intraperitoneal thymol administration (Fig. 7c, d) to the rat motor function was examined by the pole test. There were apparent deficits in motor coordination in the 6-OHDA-treated rats. The motor function impairment was severe for the rat to turn at the top of the pole (F(5,54) = 13.24, *p* < 0.001) and climb down (F(5,54) = 6.16, *p* < 0.001) compared to the control group. Intraperitoneal administration of 20 and 30 mg/kg thymol significantly exerted a recovery impact on the movement impairment.

#### Effect of thymol on beam test in 6-OHDA-lesioned rats

In the beam balance test, the number of foot faults were increased in 6-OHDA treated rats compared to the control groups in 20 cm (F(5,54) = 24.60, *p* < 0.001) and total (F(5,54) = 40.59, *p* < 0.001) beam walking. Administration of thymol significantly decreased the number of foot faults and improved motor impairment. (Fig. 7e, f).

#### Effect of thymol on rotarod test in 6-OHDA-lesioned rats

**Fig. 8:**
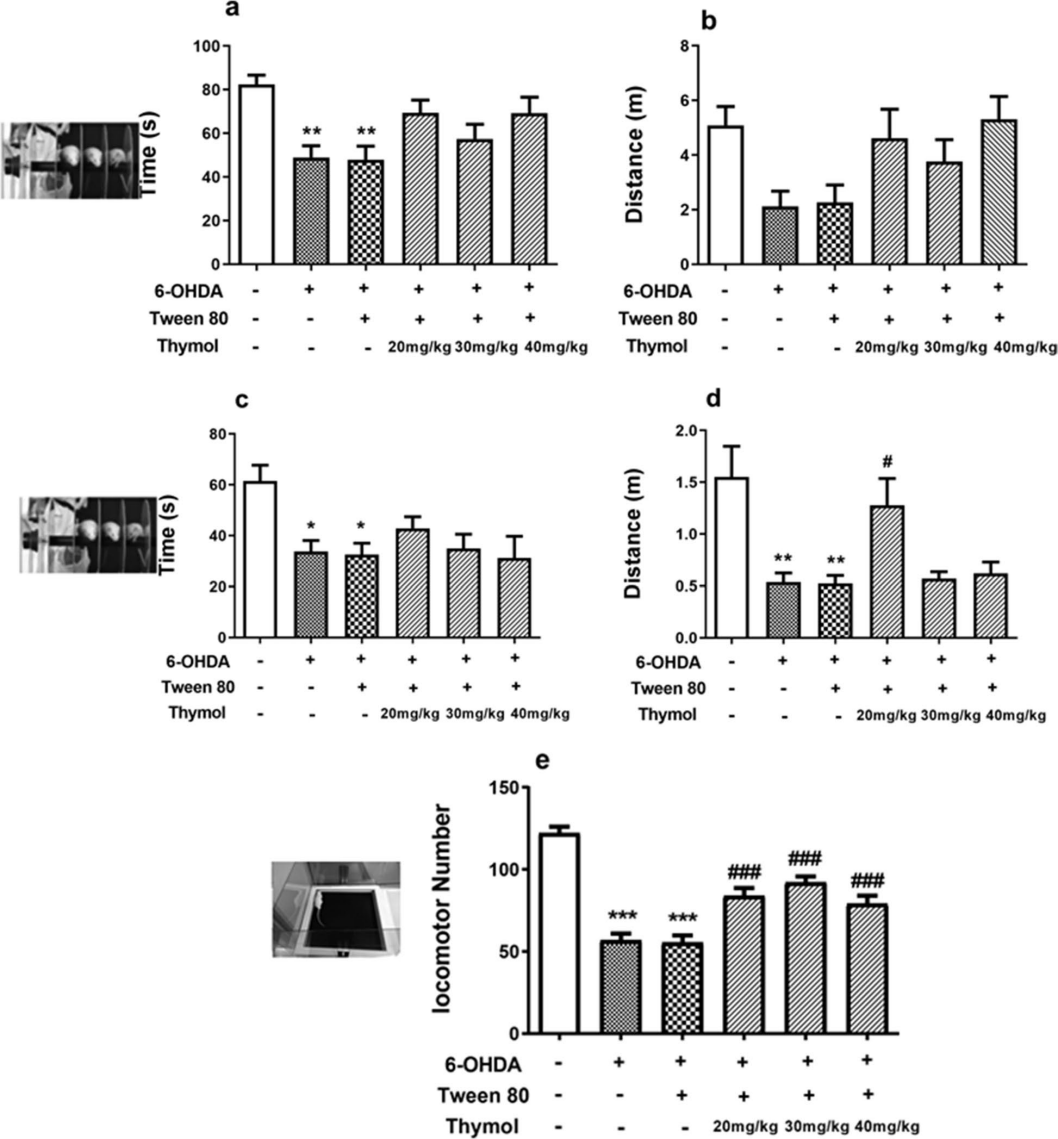
Effects of the intraperitoneal administration of thymol for 15 days on 6- OHDA-induced rotarod performance impairment. The animals were tested in the rotarod at week 2 in two settings, 5–45 rpm increase over 200 s (**a**, **b**) and 5–20 rpm increase over 20 s (**c**, **d**). This motor competency task was impaired in the 6-OHDA-lesioned animals. The thymol treatment improved the animals’ performance after the lesion. **e** In the open-field behavior, the overall distance covered was reduced by the 6-OHDA lesion and normalized by thymol. The values are presented by means ± SD for six rats in each group. *p < 0.05, **p < 0.01 and ***p < 0.001 versus the untreated control group; # *p* < 0.05 and ### *p* < 0.001 versus the 6- OHDA-treated groups

The rotarod test is most common for the measurement of motor coordination, postural balance, and bradykinesia in animal models (Park et al. 2013). Marked differences in the time spent on the rotarod either at a speed of 5–20 rpm in 20 s (F(5,42) = 3.96, *p* < 0.001) or 5–45 rpm in 200 s (F(5,42) = 5.00, *p* < 0.001) were observed in rats treated with 6-OHDA versus their counterparts in the control group (Fig. 8a,c) (Inacu et al. 2005). The exposure to 6-OHDA significantly decreased the time of stay of the rat on rod, implying there were remarkably different degrees of motor learning patterns between these animals and control rats. The subsequent injection of thymol could increase time however it was not statistically significant (*p* > 0.05). In Fig. 8b, the distance traveled at 5–45 rpm in 200 s exhibited no significant differences between thymol-treated rats and controls (*p* > 0.05) (F(5,42) = 3.24,*p* < 0.05). Although, when it came to a speed of 5–20 rpm in 20 s post-treatment administration of thymol at the dose of 20 mg/kg significantly reversed the negative effect of 6-OHDA on the travel distance (F(5,42) = 6.592, *p* < 0.001) (*p* < 0.05) (Fig. 8d).

#### Effect of thymol on open-field test in 6-OHDA-lesioned rats

Rats were placed into a 15-cm diameter open field and observed at 2 weeks post-surgery. There was a significant reduction in the number of grooming episodes after 6-OHDA lesion (*p* < 0.05) which was then restored to the control levels by thymol (*p* < 0.05 as opposed to the 6-OHDA lesion groups) (data not shown). Besides, the administration of 6-OHDA led to a significant decline in the number of rearing (*p* < 0.05) which was further compensated by thymol (*p* < 0.05 as opposed to the 6-OHDA lesion groups) (data not shown). Figure 8e indicated that locomotor activity significantly decreased in 6-OHDA treated group. All doses of thymol significantly reversed locomotor activity impairment induced by 6-OHDA (F(5,45) = 32.62, *p* < 0.001).

#### Effect of thymol on apomorphine-induced rotation in 6-OHDA-lesioned rats

15 days after the stereotaxic surgery, the animals' rotational behavior was observed as the number of ipsilateral rotations for 30 min after interaperitoneal administration of apomorphine (1 mg/kg) and one hour after the last drug administration. As shown in Fig. 7a, 6-OHDA caused an approximately four-fold increase in the number of ipsilateral rotations (*p* < 0.001). Treatment with thymol at 20 mg/kg significantly decreased apomorphine-induced rotations by 60% (F(5, 12) = 48.74, *p* < 0.001).

#### Effect of thymol on reduced glutathione test in 6-OHDA-lesioned rats

**Fig. 9:**
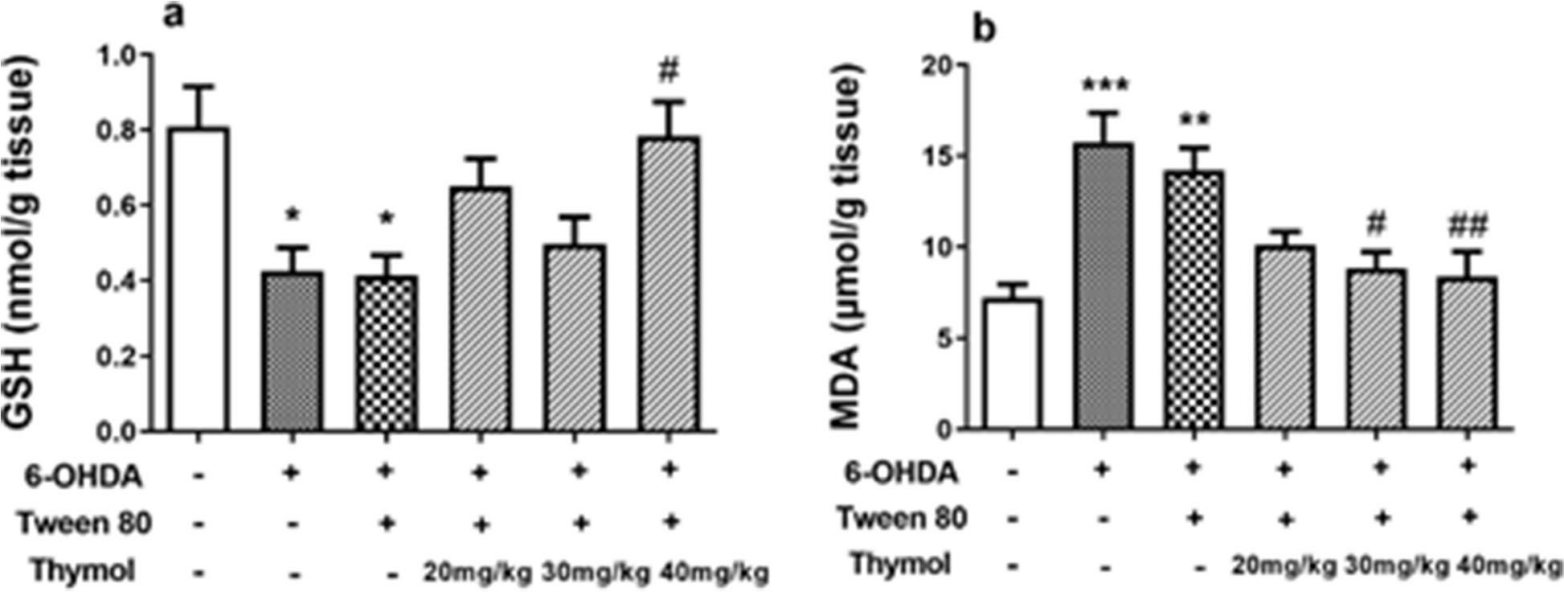
Effect of the intraperitoneal administration of thymol for 15 days on GSH (**a**) and MDA (**b**) levels in the striatum of the 6-OHDA-lesioned rats. Each measurement was done at least in triplicate, and the values are presented by means ± SD for six rats in each group. *p < 0.05, **p < 0.01 and ***p < 0.001 versus the untreated control group; # *p* < 0.05 and ###*p* < 0.01 versus the 6-OHDAtreated groups

The amount of GSH significantly attenuated in the treated group with 6-hydroxydopamine (*p* < 0.05). This result was inversed considerably in the rats exposed to thymol at 40 mg/kg (*p* < 0.05) (F(5,42) = 5.086, *p* < 0.001) (Fig. 9a).

#### Effect of thymol on lipid peroxidation in 6-OHDA-lesioned rats

The injection of 6-OHDA culminated in a significant elevation of MDA levels (*p* < 0.01) in the striatum. 15 days administration of thymol (30 and 40 mg/kg) could significantly decreased MDA levels (F(5,42) = 9.068, *p* < 0.001) (Fig. 9b).

## Discussion

In the present study, we explored the neuroprotective effect of the thymol against 6-OHDA induced neurodegeneration in PC12 cells as an in vitro 6-OHDA damaged model of PD and in rats as an in vivo 6-OHDA damaged model of PD. This medical condition is mainly characterized by loss of dopaminergic neurons and dopamine deficiency that culminate in motor disorders [38]. Dopamine resulting from dopaminergic neurons is largely carried by axons to the striatum [39]. Although dopamine replacement therapy has the capacity for the purpose of decreasing the PD symptoms, the neurodegenerative process cannot be handled in this way [38]. As for the main mechanism of the disease, oxidative stress and inflammation are well known [40]. Studies suggest that many free radicals trigger nerve apoptosis via the induction of protein and lipid peroxidation as well as DNA strands break [39]. 6-OHDA is a toxin with a highly oxidizable feature that can impair catecholaminergic neurons by means of oxidative stress. Its neurotoxicity is comparable to a molecular mechanism of DA. It has been reported that 6-OHDA participates in auto-oxidation, intra-neuronal generation of ROS, and apoptosis [41]. When 6-OHDA is oxidized by monoamine oxidase, hydrogen peroxides are generated, which further activates the generation of ROS and catecholamine quinones [42, 43]. The elevation of the reactive species levels caused by 6-OHDA culminates in the reduction of the cellular antioxidant enzymes that ultimately end up with neuronal cell death [10]. Our results showed that 6-OHDA significantly decreased the viability of PC12 cells, increased the intracellular generation of ROS, lipid peroxidation, DNA damage, and early apoptosis. These findings were corroborated previously [44–46].

Considering the role of oxidative stress in PD, the use of appropriate antioxidants can be beneficial to counteract the oxidative process. To this aim, investigations on either synthetic or natural substances with notable biological activities and multi-target mechanisms have attracted much attention in the fields of the PD treatment or prevention [47]. Thymol, identified as a natural monoterpene phenol, shows great antioxidant and neuroprotective effects [48, 49]. Similarly, we found that treatment of PC12 cells with thymol elevated the cell viability and did not have any negative influence on the cell survival even at the highest concentration of 200 μM. After 6-OHDA toxicity, post-treatment administration of thymol enhanced the viability of PC12 cells, implying the neuroprotective properties of thymol in the dopaminergic cell line. Apoptotic cell death caused by oxidative stress takes place if the ROS generation outweighs the antioxidative ability, or the endogenous antioxidant system is damaged [50]. In our study, the 6-OHDA-induced apoptosis in PC12 cells was associated with the increased intracellular ROS production. This association was also reported in Olatunji et al.’s study [45]. In this regard, the preservation of the endogenous antioxidant activity can prevent apoptosis under oxidative stress conditions [51]. The results of our study demonstrated that post-treatment administration of thymol led to a significant reduction in ROS production and cell apoptosis. Upon generation of free radicals, ROS induces lipid peroxidation and oxidizes DNA.; these are considered as leading events in the etiopathogenesis of PD [52]. We also observed that thymol could significantly inhibit lipid peroxidation in TBARS assay.

It has been documented that 6-OHDA leads to the degeneration of nigrostriatal dopaminergic neurons as well as motor and non-motor impairments in common with those experienced by PD patients [53–55]. Our findings were consistent with these changes so that the administration of 6-OHDA caused motor deficits as evidenced by increased rotations. This observation was confirmed by other studies [56–58]. The unilateral lesion of the nigrostriatal dopaminergic system associated with exposure to 6-OHDA lowers dopamine levels in the striatum but augments dopamine post-synaptic receptors on the same side. Such alterations result in a motor asymmetry examined by dopamine agonists like apomorphine [59]. Accordingly, apomorphine-induced rotations in 6-OHDA-lesioned rats can be indicative of the nigrostriatal dopamine depletion. The PD symptoms develop if there is a loss around 60–80% in the dopamine level and 50–60% in the dopaminergic neurons in the substantia nigra [60]. Similar to the previous findings [61, 62], 6-OHDA-lesioned rats in our study displayed motor alterations in the catalepsy tests. Likewise, impairments in motor performances as found in the beam walking, rotarod, pole, and open-field tests were clearly observed in the 6-OHDA-treated rats. These results were also reported by other studies [56, 61, 63, 64].

Further investigations on 6-OHDA-lesioned rats indicated that thymol ameliorated 6-OHDA-induced rotational behavior. Consistent with our findings on thymol, the positive contribution of other antioxidants like paeoniflorin [57], L-linalool [56], resveratrol [63], and carvacrol [64] were indicated. However, Haddadi et al. did not observe any positive effect for carvacrol on apomorphine-induced rotations in rats [58]. Consistent with our more recent study on carvacrol [64], treatment with the certain dose of thymol could effectively improve catalepsy as evidenced by significantly decreased time on the bar in the catalepsy test and movement impairment as evidenced by the significantly reduced pole time. A potential drug for the treatment of PD is expected to obtain behavioral benefits in two ways: direct activation of the dopaminergic receptor or protection of the dopaminergic neurons against 6-OHDA toxicity [65]. In this regard, we observed that the administration of thymol into 6-OHDA-lesioned rats improved motor dysfunction in the rotarod test and resulted in longer latency for initiation of narrow beam test besides total time required for crossing. In other words, the lesioned rats delayed initiating the task with a lower speed while crossing the beam. Such observations indicated the sign of bradykinesia or akinesia [66]. These findings were in line with those obtained in the studies by Manouchehrabadi et al. [64], Nataraj et al. [67], and Khan et al. [63]. In a different PD model, Rekha also reported the effectiveness of an acyclic monoterpene alcohol, geraniol, with antioxidant potential in enhancing severe motor deficits in MPTP (1-methyl-4-phenyl-1, 2, 3, 6-tetrahydropyrindine)-treated mice [68]. In addition, thymol significantly prevented the decreased locomotor activity observed in the open-field test as an indicator of motor incoordination. The existing evidence has confirmed our results [56, 64]. Moreover, the neuroprotective effect of thymol is supported by its positive contribution to 6-OHDA-induced oxidative stress in a PD rat model. 6-OHDA resulted in the degeneration of dopaminergic neurons through the production of free radicals and then the induction of oxidative stress. In the brain, GSH is an important antioxidant that neutralizes ROS. Our findings exhibited the significant decrease in the GSH level in the 6-OHDA lesioned group, which further showed the significant restoration of GSH upon treatment with thymol. The perturbation of endogenous antioxidant defenses, like GSH, has been well studied in the brain tissues of the PD models [69]. The detoxification of free radicals by means of antioxidants like thymol carries inhibitory impacts on cell damage, which, in turn, prohibits the progress of lipid peroxidation [70]. Another reliable marker of lipid peroxidation is MDA that significantly elevated in our 6-OHDA lesioned group but considerably reduced in our thymol-treated group. The measurement of oxidative stress markers in our study revealed the strong role of thymol in improving the antioxidant defense system as well as mitigating the toxicity of 6-OHDA. The main cause underlying the potent antioxidant and free radical scavenging features of thymol is related to its phenolic content that can afford to absorb or neutralize free radicals and enhance endogenous antioxidants against the tremendous impacts of free radicals induced oxidative stress [71]. The effective role of natural antioxidants in protection against lipid peroxidation caused by 6-OHDA in the PD rats were was found previously [56, 63, 64, 70]. The current study findings exhibited that 6-OHDA triggered a considerable elevation of oxidative stress-related damages in the cellular and animal models of PD, compared to controls. Nevertheless, post-treatment with thymol could successfully recover those negative effects, which imply the potent antioxidant effects of thymol.

## Data Availability

The datasets used for the current study are available from the corresponding author upon request.
